# Dexmedetomidine decreases cerebral hyperperfusion syndrome incidence following mechanical thrombectomy in acute ischemic stroke: a double-blind, randomized controlled trial

**DOI:** 10.3389/fneur.2025.1680268

**Published:** 2025-09-17

**Authors:** Ziwen Gao, Rihua Zhou, Benling Sang, Guolin Gao, Shu Li, Jiaxin Li

**Affiliations:** Department of Anaesthesiology, Civil Aviation General Hospital, Beijing, China

**Keywords:** dexmedetomidine, cerebral hyperperfusion syndrome, acute ischemic stroke, mechanical thrombectomy, postoperative pain scores

## Abstract

**Background:**

Cerebral hyperperfusion syndrome (CHS) is a serious complication that can follow intravascular mechanical thrombectomy for acute ischemic stroke (AIS). Dexmedetomidine (Dex), a selective *α*₂-adrenoceptor agonist used as a sedative, has known neuroprotective effects in ischemic cerebral injury. This double-blind, randomized, placebo-controlled clinical trial (ChiCTR 2500105088) aimed to evaluate the preventive impact of low-dose Dex on CHS after AIS.

**Methods:**

Patients with AIS and anterior circulation occlusion scheduled for endovascular mechanical thrombectomy from August 2023 to October 2024 were included. The occluded vessels were the internal carotid artery intracranial portion, M1, or M2 segments of the middle cerebral artery. After obtaining informed consent, patients were randomly allocated to two groups: one group (*n* = 70) received intravenous Dex with a 10-min preoperative loading dose of 0.5 μg/kg, followed by postoperative maintenance infusion at 0.1 μg/kg/h until 72 h postoperatively. The other group (*n* = 71) received an equal volume of placebo (normal saline) via the same intravenous route and schedule. The principal outcome was the occurrence of CHS evaluated through the seventh day post-operation. Subsidiary outcomes comprised the National Institutes of Health Stroke Scale (NIHSS) score within 24 h post-operation, Modified Rankin Scale (mRS) scores at discharge, within 30 days and 90 days post-operation, the duration of ICU stay, total hospital stay length, and the 30-day all-cause mortality rate.

**Results:**

A statistically significant reduction in the occurrence of CHS was observed in the Dex group relative to the placebo group: among 70 patients in the Dex group, only 2 cases of CHS were identified (2.9%), whereas 10 cases occurred in the placebo group (14.1%) from a total of 71 patients. This difference was confirmed by both odds ratio (OR: 0.203; 95% confidence interval [CI]: 0.046–0.893; *p* = 0.017) and hazard ratio (HR: 0.194; 95% CI: 0.043–0.887; *p* = 0.018) analyses. Additionally, the Dex group showed significantly lower postoperative pain scores assessed via the Numeric Rating Scale (NRS) on postoperative day 1 and day 3 compared with the placebo group (*p* < 0.0001).

**Conclusion:**

Dex significantly reduced 7-day CHS occurrence after mechanical thrombectomy in AIS patients and lowered postoperative pain scores.

**Clinical trial registration:**

www.chictr.org.cn, identifier ChiCTR 2500105088.

## 1 Introduction

Endovascular mechanical thrombectomy is now the mainstay intervention for acute ischemic stroke (AIS), effectively ameliorating neurological deficits in patients (1). While reperfusion substantially enhances cerebral perfusion, the compromised autoregulation in the injured brain tissue renders it susceptible to cerebrovascular edema and intracranial hypertension. This cascade of events can culminate in the onset of cerebral hyperperfusion syndrome (CHS) (2). Prior research has established that patients with acute-onset anterior circulation arterial infarction exhibit impaired autoregulation of ipsilateral cerebral blood flow during the initial 10 days post-stroke (3). Carotid artery stenting and carotid endarterectomy were initially associated with CHS. More recently, CHS has also been recognized as a possible complication of intravenous thrombolysis and endovascular mechanical interventions for AIS (4, 5). A prospective study on individuals diagnosed with acute middle cerebral artery infarction revealed a 34% incidence of CHS (4). The mean velocity index of the middle cerebral artery, as assessed via transcranial Doppler (TCD) ultrasound, indicates that an increase in this parameter following stroke thrombectomy is associated with an elevated risk of cerebral hemorrhage (6). Consequently, close monitoring of middle cerebral artery blood flow velocity is warranted after stroke thrombectomy procedures. Presently, aside from improving perioperative monitoring and stabilizing hemodynamics, there are no established preventive or therapeutic strategies for CHS. Dexmedetomidine (Dex) is a kind of sedative, widely used in surgical anesthesia process, with neuroprotective effect. The neuroprotective mechanisms of this compound include modulation of neurotransmitters, inhibition of inflammatory responses, attenuation of neuronal apoptosis through diverse cellular signaling pathways, and regulation of mitochondrial function, as demonstrated in animal models of ischemia–reperfusion injury (7–9). Initial case studies have indicated the efficacy of Dex in managing CHS following carotid endarterectomy (10). A retrospective study indicated that in patients with moyamoya disease, Dex did not reduce the incidence of CHS but shortened its duration postoperatively. This effect may be attributed to the patients’ prolonged cerebral infarction or hypoperfusion state prior to surgery, during which collateral circulation or vasodilation had developed, counteracting the vasoconstrictive effects of Dex. Additionally, Dex was administered only briefly during the procedure (11). Dex can induce adverse effects such as dose-dependent bradycardia and hypotension (12), thereby restricting its broad utilization in patients with AIS. Research indicates that administering a low dose of Dex postoperatively (0.1 μg/kg/h) can enhance sleep quality without altering sedation scores, decrease postoperative delirium, and have negligible effects on hemodynamics (13). The central aim of the present study is to investigate the potential neuroprotective impact of Dex on individuals who have experienced AIS. Specifically, the study seeks to determine if administering Dex via low-dose continuous infusion can decrease the occurrence of postoperative CHS in patients with AIS.

## 2 Methods

### 2.1 Study design

A prospective randomized, double-blind, placebo-controlled trial was performed at the Department of Interventional Neurology and Department of Anesthesiology, Civil Aviation General Hospital, Beijing, China, between August 2023 and October 2024. The primary aim of the present study was to evaluate the superiority of the investigational intervention in perioperative neurological management. This study was approved by the Institutional Review Board of Civil Aviation General Hospital (approval number: 2022-L-K-48). Written informed consent was obtained from each patient or their legally authorized representative prior to enrollment (Supplementary materials).

### 2.2 Participants

Patients undergoing endovascular mechanical thrombectomy at Civil Aviation General Hospital from August 2023 to October 2024 were screened based on specific criteria. Inclusion criteria encompassed an age of 18 years or older; internal carotid artery occlusion or middle cerebral artery occlusion involving the M1 and M2 segments, for which endovascular mechanical thrombectomy under general anesthesia was indicated; onset within 6 h, and a pre-onset Modified Rankin Scale (mRS) score ≤ 2. Exclusion criteria comprised Alberta Stroke Program Early CT Score (ASPECTS) ≤ 6 pre-onset, intracranial hemorrhage, cerebral infarction area > 1/3 of the anterior circulation blood-supply area, or arterial dissection; history of contrast-agent allergy, lactation, or contraindications for endovascular treatment; active bleeding or bleeding tendency history; severe heart, lung, or kidney dysfunction; inadequate operative outcomes (residual stenosis rate > 30%, thrombus detachment); and history of ipsilateral carotid endarterectomy or restenosis.

### 2.3 Randomization and blinding

An independent biostatistician, uninvolved in subsequent data handling and statistical analysis, generated a 1:1 randomization sequence using IBM SPSS Statistics 23.0 software. This sequence was placed in numbered envelopes and stored at the study site until the trial’s conclusion. Throughout the study, consecutive AIS patients were allocated to either the Dex group or the placebo group based on the randomization sequence and subsequently received the designated intervention.

Dedicated pharmacy nurses were designated as drug administrators for this study, who were responsible for preparing and dispensing study drugs according to the randomization sequence. Throughout the study, healthcare staff including surgical and anesthesia teams, outcome evaluators, and patients remained masked to the treatment allocation. In urgent situations such as acute patient deterioration or clinical emergencies, the attending physician was authorized to modify or discontinue drug administration or request unblinding of the treatment assignment based on clinical necessity, with all relevant details documented meticulously. Notably, no unblinding events occurred during the study period.

### 2.4 Procedures

Study drugs were prepared by dedicated pharmacy nurses not involved in outcome assessment, according to the randomization sequence. For the Dex group, Dex hydrochloride (200 μg/2 ml) was diluted with normal saline to a total volume of 200 ml, achieving a final concentration of 1 μg/ml. For the placebo group, an equal volume of normal saline (200 ml) was prepared as the placebo. Both the Dex solution and placebo were dispensed in identical transparent containers with indistinguishable appearance and specifications to maintain blinding (Jiangsu Hengrui Medicine Co., Ltd., Jiangsu, China). Upon arrival in the operating room, prior to anesthesia induction, the study drug was administered via intravenous infusion at a volume of 0.5 ml/kg over 10 min (corresponding to an actual dose of 0.5 μg/kg in the Dex group). Postoperatively, continuous infusion of the study drug was maintained at a rate of 0.1 ml/kg per hour (corresponding to an actual dose of 0.1 μg/kg/h in the Dex group) until 72 h postoperatively. Preoperative assessment included the collection of baseline patient demographic and clinical data, while invasive hemodynamic monitoring via radial artery catheterization was established under local anesthetic administration prior to the procedure.

For anesthesia induction, etomidate was intravenously administered at doses ranging from 0.2 to 0.6 mg/kg, sufentanil at 0.1 to 0.5 μg/kg, and rocuronium bromide at 0.6 to 1 mg/kg. For anesthesia maintenance, propofol was continuously infused at a rate of 4 to 12 mg/kg/h, and remifentanil was administered intravenously at 0.1 to 0.3 μg/kg/min. Prior to opening the occluded vessel, intraoperative mean arterial pressure (MAP) was rigorously controlled within ±10% of the preoperative baseline. Norepinephrine was delivered intravenously at a continuous rate of 2–6 μg/kg/h if needed.

The patient was positioned supine, and following general anesthesia, the groin area was disinfected and draped with sterile towels in accordance with aseptic principles. Access was gained via the right femoral artery. Using the Seldinger technique, a successful puncture was achieved, followed by systemic heparinization. An 8F arterial sheath was then inserted. Through this sheath, a 5F VER catheter and a Glidewire were advanced to the aortic arch and cerebral vessels for angiographic examination to pinpoint the site of vascular occlusion. A 6F delivery catheter was coaxially inserted and connected to a high-pressure drip system. Using the roadmap, a microguidewire and microcatheter navigated through the thrombus to reach the distal end of the occluded segment. Once the microcatheter was confirmed to be in the true lumen, a mechanical thrombectomy device was positioned. Following the removal of the microguidewire and microcatheter, thrombectomy was performed using negative pressure aspiration or by retrieving the thrombectomy stent. The procedure was repeated as needed until forward blood flow achieved mTICI grade 2b or higher (14). The operator chose the thrombectomy approach-stent retrieval, aspiration, or a combination-based on the patient’s vascular condition and thrombus characteristics (15). Following successful recanalization, MAP was maintained between 70 and 105 mmHg (16). Nicardipine (0.5–6 μg/kg/min) could be administered if necessary. Postoperatively, all patients underwent computed tomography (CT) to assess for cerebral hemorrhage. In the absence of hemorrhage, tirofiban antiplatelet therapy could commence within 24 h. Endotracheal tubes were removed when patients met the criteria for extubation, after which they were transferred to the post-anesthesia care unit for postoperative monitoring, with a minimum duration of 6 h.

### 2.5 Postoperative auxiliary examinations

Routine non-contrast cranial CT was performed within 24 h postoperatively to exclude cerebral hemorrhage, subarachnoid hemorrhage, and detect progression of cerebral infarction or other relevant findings. Additionally, in cases of new or worsening postoperative neurological deficits, immediate repeat non-contrast cranial CT was warranted to identify the underlying cause.

Using a 1.6 MHz probe from the EMS-9 PB TCD system (Shenzhen Delica Medical Devices Co., Ltd., Shenzhen, China), we monitored the mean flow velocity (MFV) of the middle cerebral artery (MCA) on both the ipsilateral (recanalized) and contralateral sides via the temporal window at these intervals: d0 (within 2 h post-surgery), d1 (24 h), d2 (48 h), d3 (72 h), d4 (96 h), d5 (120 h), d6 (144 h), and d7 (168 h). The MCA MFV index was calculated as the ratio of MFV on the recanalized side to the contralateral side. A risk of cerebral hyperperfusion was indicated if this index exceeded 1.3 or if there was a significant increase in MCA MFV on the recanalized side compared to the previous measurement (6).

### 2.6 Outcome measures

The study’s main outcome measure was the occurrence of postoperative CHS within 7 days postoperatively. CHS is defined as the presence of one or more of the following clinical manifestations: headache, ophthalmic or facial pain, vomiting, focal neurological deficits, intracerebral or subarachnoid hemorrhage, and malignant hypertension (systolic blood pressure > 180 mmHg and/or diastolic blood pressure > 100 mmHg). These symptoms must be confirmed to be non-secondary to recurrent cerebral infarction, surgical complications, or other systemic etiologies, with TCD findings indicating the presence of hyperperfusion (2).

Secondary indicators comprised the National Institute of Health Stroke Scale (NIHSS) score at 24 h post-operation, the mRS scores at discharge, within 30 and 90 days post-operation, the Richmond Agitation-Sedation Scale (RASS) score on the third day post-operation, the Numerical Rating Scale (NRS) pain scores on the first and third days post-operation, the duration of ICU stay, the overall hospitalization period, and the 30-day all-cause mortality rate.

During the study, data on MAP, heart rate (HR), and SPO2 were gathered from patients at various time points: upon arrival in the operating room (T0), post-anesthesia induction (T1), pre-skin incision (T2), 5 min post-operation commencement (T3), post-reperfusion (T4), end of surgery (T5), 30 min post-operation (T6), 1 h post-operation (T7), and at 8:00 AM on the 1st, 3rd, and 7th postoperative days. Furthermore, the duration from entering the operating room to achieving vascular reperfusion was documented (17).

Adverse events were documented and managed as follows: Bradycardia was characterized by a HR below 55 beats per minute or a reduction exceeding 20% from baseline; hypotension was characterized as either a systolic blood pressure < 95 mmHg or a reduction > 20% relative to baseline values; tachycardia was characterized by HR > 100 beats per minutes or elevation > 20% compared with baseline measurements. Hypertension was characterized as a systolic blood pressure over 160 mmHg or an elevation exceeding 20% relative to baseline values; hypoxemia was specified as a peripheral SPO2 below 90% or a reduction exceeding 5% in absolute value from baseline measurements. If any of the above-mentioned situations occurred, interventions involved modifying the infusion rate of the investigational drug or administering appropriate symptomatic treatment. Notably, no patients discontinued participation in the study due to severe adverse events.

### 2.7 Statistical methods

This study employed a superiority trial with the occurrence of postoperative CHS as the primary outcome measure. Based on pre-trial data, the anticipated incidence rates of postoperative CHS in the two groups were 3 and 18%, respectively. The one-sided *α* was predetermined as 0.025, with a power of 80%. Utilizing PASS 14.0 software for calculations, a sample size totaling 65 participants per group was determined, accounting for a 10% anticipated dropout rate. Overall, 145 patients were enrolled for the study.

Statistical analyses were executed utilizing SPSS 22.0 software. Quantitative variables that conformed to a normal distribution were presented as *x̄*±*s* and subjected to comparison using the independent-samples t-test. Repeated measures analysis of variance was applied to compare HR and MAP between the two groups at various time points spanning the intraoperative and postoperative phases. The significance threshold was established at *α* = 0.05, consistent with standard statistical conventions for hypothesis testing. Quantitative variables exhibiting a non-normal distribution were summarized as median and interquartile range, which were subjected to intergroup comparisons via the Wilcoxon rank-sum test. Categorical variables were recapitulated as percentages and subjected to intergroup comparisons via *χ*^2^ test or Fisher’s exact test. The occurrence of CHS within 7 days post-operation was examined through survival analysis; group distinctions were evaluated using the Log-Rank test, and efficacy was evaluated via the Cox proportional hazards model. All statistical tests were two-tailed, with *p* < 0.05 indicating statistical significance.

## 3 Results

### 3.1 General data

**Figure 1:**
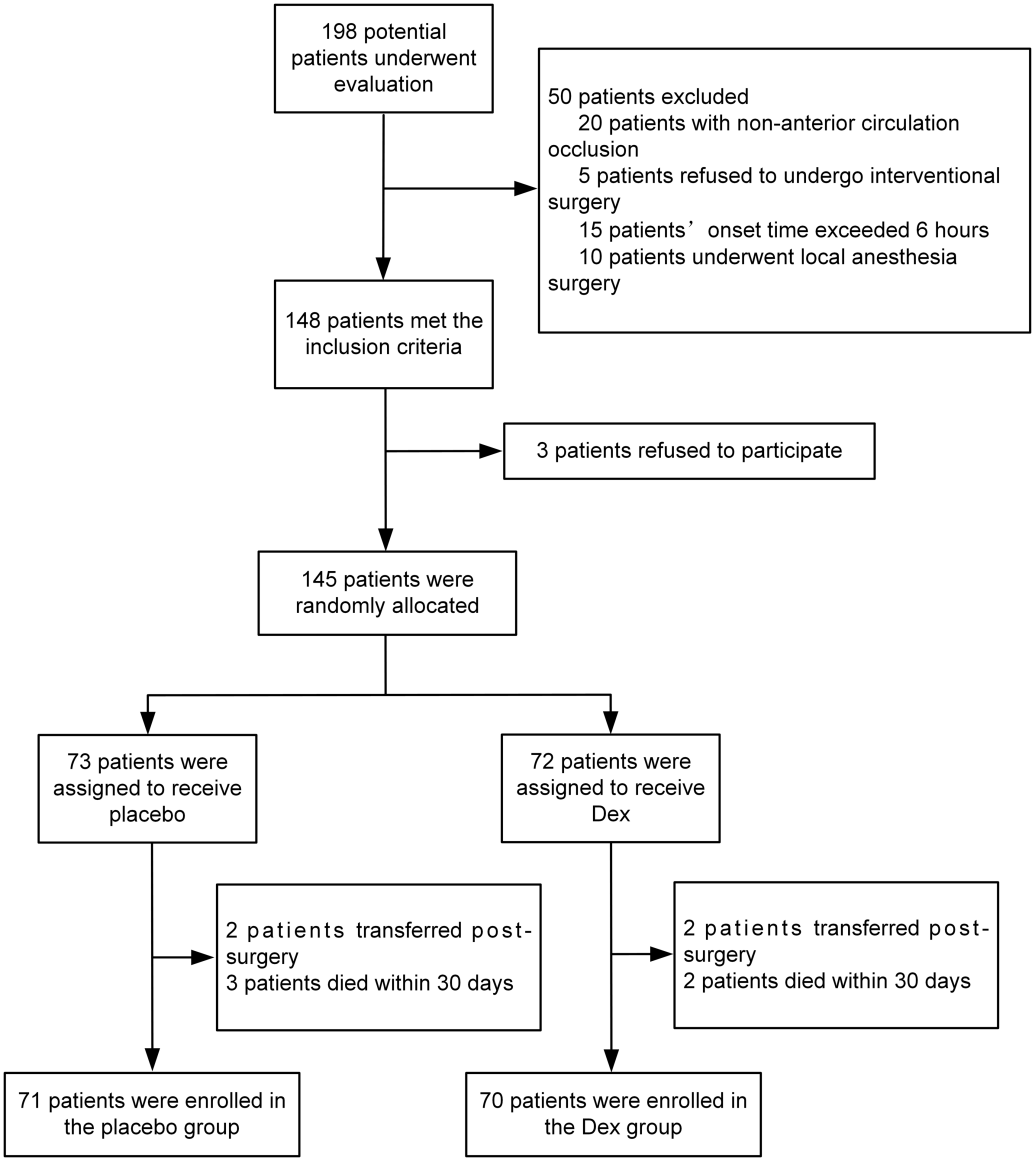
Flow chart of patient enrollment.

**Figure 2:**
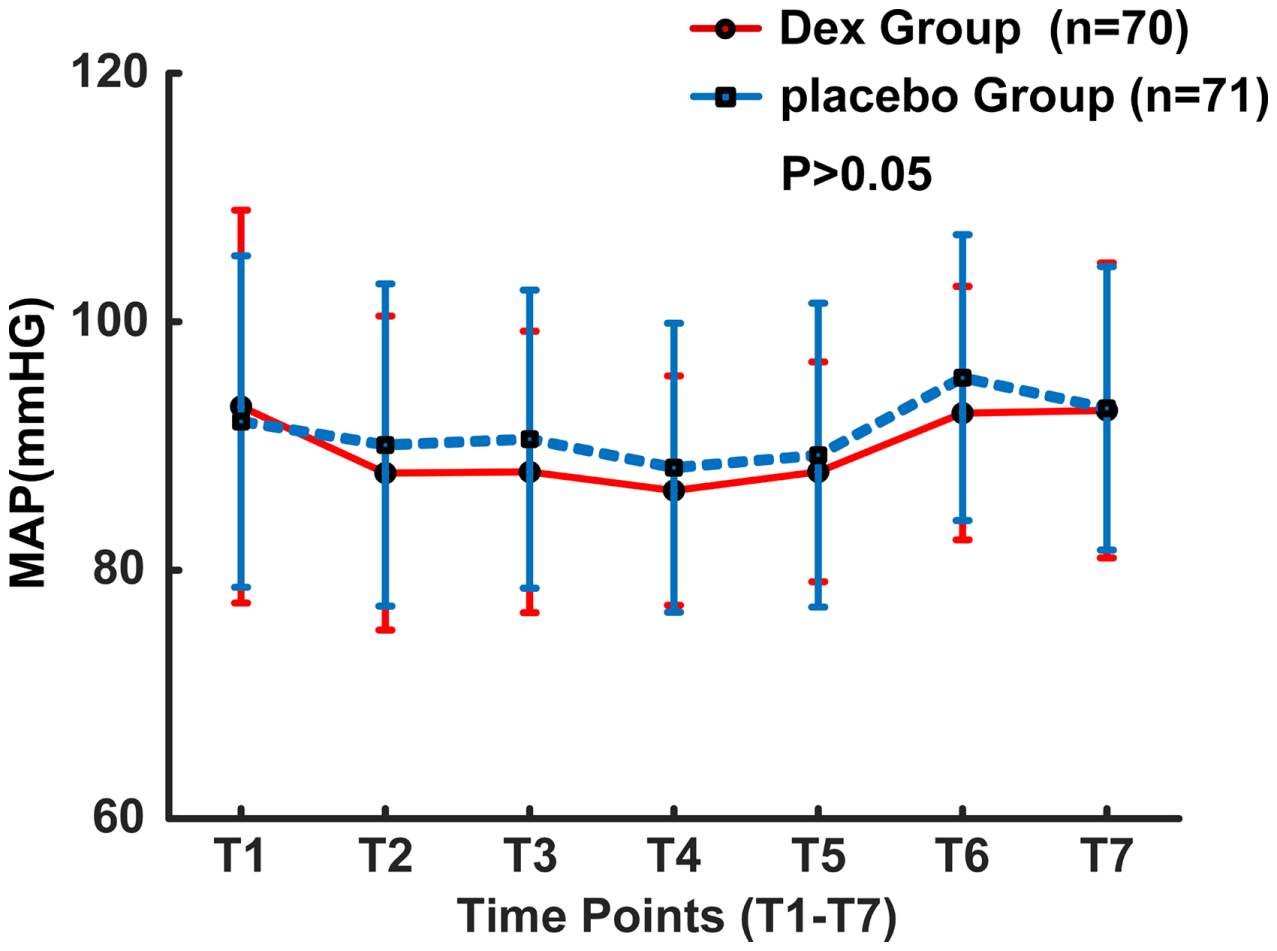
MAP changes during the operation.

**Figure 3:**
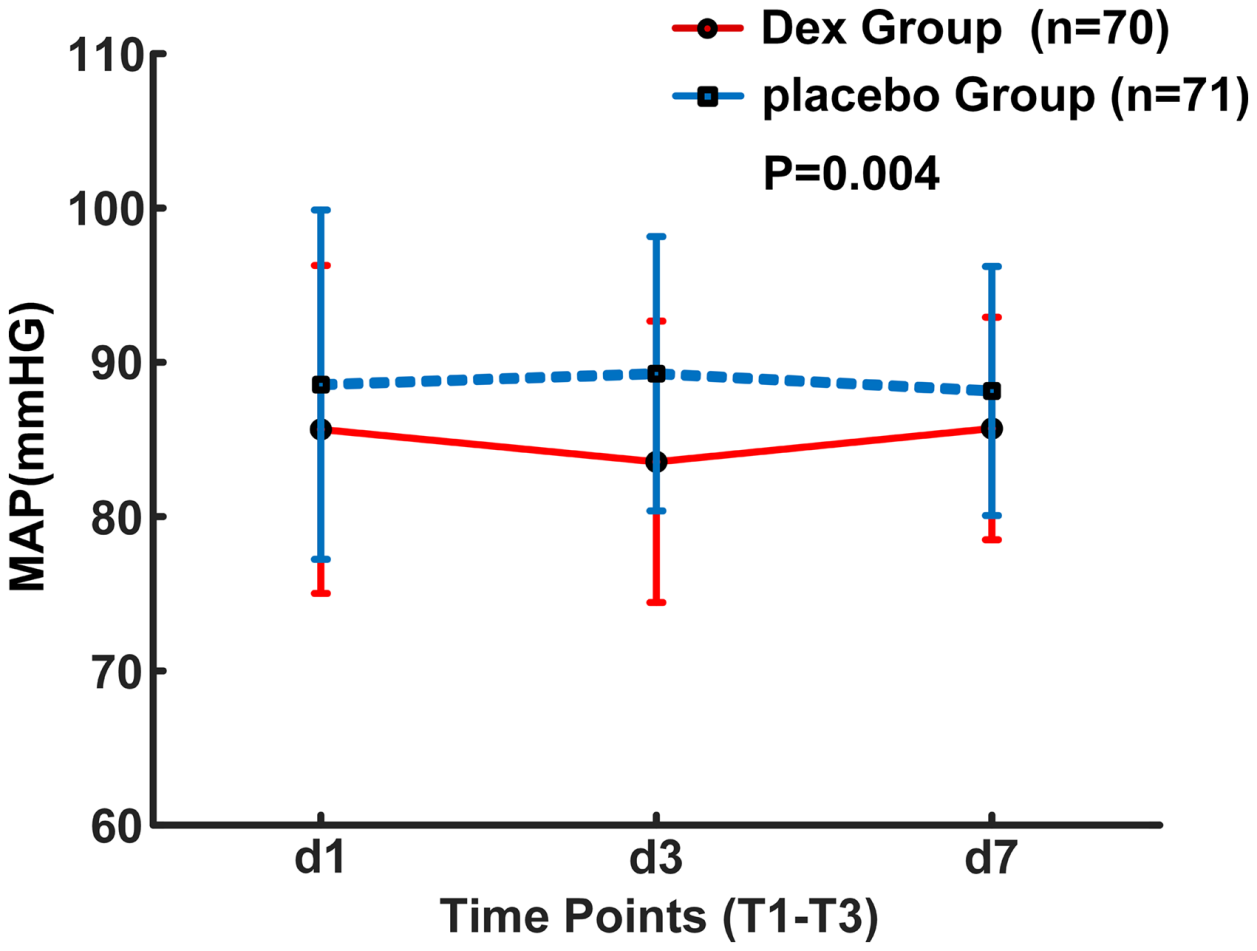
Postoperative MAP changes over the first 3 days.

From August 2023 to October 2024, a total of 198 patients who had undergone interventional endovascular mechanical thrombectomy at Civil Aviation General Hospital were initially screened. Among them, 53 patients were excluded, with reasons specified as follows: 20 patients with non-anterior circulation occlusion; 5 patients refused to undergo interventional surgery; 15 patients’ onset time exceeded 6 h; 10 patients underwent local anesthesia surgery; 3 patients refused to participate. 145 individuals were ultimately included in the study and randomly allocated to either the Dex group (*n* = 72) or the placebo group (*n* = 73). 4 patients transferred post-operation, with 2 from each group, resulting in a final analysis cohort of 141 patients, with 70 in the Dex group and 71 in the placebo group (Figure 1). Baseline characteristics and perioperative variables showed no statistically significant between-group differences across the two study cohorts (*p* > 0.05, Table 1). Of note, perioperative blood pressure measurements were consistently lower in the Dex group relative to the placebo group during the perioperative period. There were no significant intergroup differences in the changes of intraoperative MAP at different time points between the two groups (*F* = 0.87, *p* > 0.05, Figure 2). Postoperatively, MAP within three days after Dex administration was lower than that in the placebo group, and the difference was statistically significant (*F* = 8.687, *p* = 0.004, Figure 3).

**Table 1:** Baseline indicators and perioperative variables.

Variable	Dex (*n* = 70)	Placebo (*n* = 71)	*p*-value
Preoperative			
Age (years)	62.09 ± 10.58	65.30 ± 12.79	0.107
BMI (kg·m^−2^)	25.23 ± 3.57	24.60 ± 3.12	0.270
Male, *n* (%)	52 (74.28%)	48 (67.61%)	0.382
Smoking, *n* (%)	33 (47.14%)	33 (46.48%)	0.937
Medical history, *n* (%)			
Coronary heart disease	18 (25.71%)	14 (19.72%)	0.427
Diabetes mellitus	33 (47.14%)	23 (32.39%)	0.086
Hypertension	56 (80.00%)	62 (87.32%)	0.263
Previous stroke, *n* (%)	29 (41.43%)	26 (36.61%)	0.607
Occlusion site (right), *n* (%)	25 (35.71%)	33 (46.47%)	0.232
Preoperative mRS (score)	3 (2–4)	3 (2–4)	0.942
Pre-operative NIHSS(score)	7.5 (7–11)	10 (7–14)	0.161
ASPECTS	8 (8–9)	8 (7–9)	0.530
ASITN/SIR	2 (2–2)	2 (2–2)	0.635
HR (bpm)	75.37 ± 12.29	75.97 ± 10.78	0.758
MAP (mmHg)	104.87 ± 13.29	101.76 ± 12.22	0.150
Duration of operation (min)	56.50 (40.0–85.0)	59.00 (40.0–100.0)	0.526
Vasoactive drugs during procedure			
Norepinephrine, *n* (%)	13 (18.57%)	10 (14.08%)	0.502
Atropine, *n* (%)	8 (11.43%)	10 (14.08%)	0.802
Nicardipine, *n* (%)	30 (42.86%)	24 (33.80%)	0.301
Anesthetics			
Sufentanil (μg)	17.80 ± 5.96	17.93 ± 6.45	0.902
Propofol (mg)	510.40 ± 174.70	515.80 ± 171.40	0.851
Remifentanil (μg)	864.79 ± 318.79	855.28 ± 300.67	0.856
Etomidate (mg)	19.23 ± 4.83	19.00 ± 5.08	0.785

Data are mean ± SD, median (interquartile range) or number (%). Dex, dexmedetomidine; BMI, body mass index; mRS, Modified Rankin Scale; NIHSS, National Institute of Health Stroke Scale; MAP, mean arterial pressure.

### 3.2 Prognosis comparison

**Figure 4:**
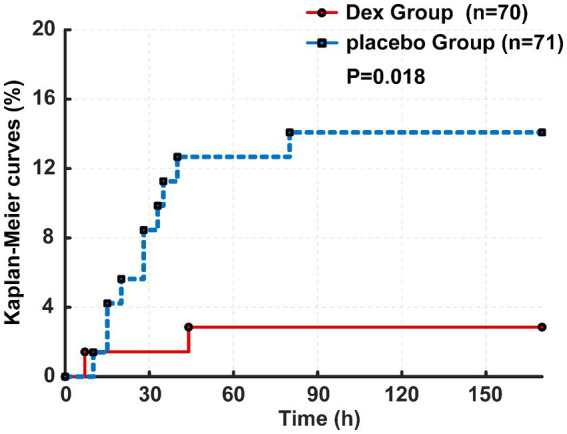
Kaplan–Meier curves of CHS between two groups.

Primary observation indicators show that within the 7-day perioperative period, the incidence of CHS was documented as 2.9% in the Dex group, corresponding to 2 cases among 70 enrolled patients, whereas the placebo group exhibited a CHS incidence of 14.1%, with 10 cases identified among 71 patients. The hazard ratio for CHS occurrence was 0.194 with a 95% confidence interval of 0.043–0.887 (*p* = 0.018, Figure 4).

The incidence of CHS in patients treated with Dex exhibited an odds ratio of 0.203, with a 95% confidence interval ranging from 0.046 to 0.893 (*p* = 0.033, Table 2). Secondary outcomes did not reveal statistically significant differences between the Dex group and the placebo group for hospital stay duration, ICU stay duration, NIHSS score at 24 h post-operation, mRS score at discharge, within 30 and 90 days postoperative, and 30-day all-cause mortality rate. Furthermore, the Dex group exhibited significantly lower NRS pain scores as opposed to the placebo group on 24 and 72 h postoperative (*p* < 0.05, Table 2). Upon cessation of the study drug infusion, no statistically significant difference was noted in the RASS scores between the two groups (*p* > 0.05, Table 3).

**Table 2:** Study outcomes and post-operative NRS score.

Variable	Dex group (*n* = 70)	Placebo group (*n* = 71)	*p*-value
The overall incidence of CHS	2 (2.86%)	10 (14.08%)	0.016
Length of hospital stay (day)	9 (6–12)	9 (7–12)	0.629
Length of ICU stay (day)	1.50 (1–2)	1 (0–3)	0.246
NIHSS (score) at 24 h postoperative	1 (2–7)	0 (2–7)	0.855
mRS score			
At discharge	2 (2–3)	2 (2–3)	0.839
At 30 days postoperative	2 (2–3)	2 (2–3)	0.172
At 90 days postoperative	2 (2–3)	2 (2–3)	0.237
30-day all-cause mortality	2 (2.86%)	3 (4.22%)	1.000
NRS for pain at 24 h postoperative (score)	1 (1–1)	2 (1–2)	0.000
NRS for pain at 72 h postoperative (score)	0 (0–0)	0 (0–1)	0.000

Data are median (interquartile range) or number (%). Dex, dexmedetomidine; CHS, cerebral hyperperfusion syndrome; ICU, intensive care unit; NIHSS, National Institute of Health Stroke score; mRS, modified Rankin Scale; NRS, numeric rating scale.

**Table 3:** Comparison of adverse events.

Variable	Dex group (*n* = 70)	Placebo group (*n* = 71)	*p*-value
RASS score at 72 h postoperative (scale)	0 (0–0)	0 (0–0)	0.628
Post-operative cerebral infarct, *n* (%)	1 (1.43%)	1 (1.41%)	1.000
Post-operative cerebral hemorrhage, *n* (%)	0 (0%)	1 (1.41%)	1.000
Post-operative hypertension, *n* (%)	1 (1.43%)	1 (1.41%)	0.992
Post-operative tachycardia, *n* (%)	0 (0.0%)	6 (8.45%)	0.013
Post-operative bradycardia, *n* (%)	0 (0.0%)	1 (1.41%)	0.319

Intraoperative dexmedetomidine was administered to all patients in the Dex group, while no such treatment was given to those in the placebo group. Data are presented as median (interquartile range) or number (%). RASS, Richmond Agitation Sedation Scale; Dex, dexmedetomidine.

### 3.3 Adverse events comparison

There was no significant difference observed between the two groups concerning the use of vasoactive medications for abnormal blood pressure and HR (*p* > 0.05, Table 1). The Dex group exhibited a notably lower occurrence of sinus tachycardia as opposed to the placebo group (*p* < 0.05, Table 3). No substantial difference was noted in the frequency of postoperative minor stroke and cerebral hemorrhage between the two groups.

## 4 Discussion

Dex is a potent α2-adrenergic receptor agonist widely employed as a sedative in clinical settings. Its precise neuroprotective properties against ischemic brain injury have been well-established, with supporting evidence from animal studies (18–23). Current studies on perioperative sedation in patients with AIS have been predominantly focused on comparisons of intraoperative anesthetic regimens (23, 24), with no definitive reports on postoperative sedation. This may stem from the fact that AIS patients require dynamic assessment of neurological status, while sedation could mask signs of disease deterioration such as altered consciousness or new-onset neurological deficits. To avoid such scenarios, based on the controllable central and peripheral sympathetic inhibitory effects of Dex, the present study adopted a regimen consisting of preoperative single-dose intravenous bolus of Dex combined with postoperative low-dose continuous infusion of Dex. Post-infusion assessment of sedation depth revealed that postoperative Dex infusion at 0.1 μg/kg/h did not exert significant sedative effects in patients, nor did it interfere with real-time neurological status assessment during the infusion period, which constitutes a key innovation of the present study. This study demonstrates that administering prophylactic low-dose Dex before and after surgery effectively decreases the occurrence of postoperative CHS within a 7-day period in patients with AIS. We posit that this outcome is intricately connected to the neuroprotective properties of Dex. CHS is associated with the partial compromise of the blood–brain barrier, compromised cerebral autoregulation, and a sudden surge in cerebral blood flow following vascular recanalization in individuals experiencing ischemic stroke (2, 25). Several studies have demonstrated that Dex has the potential to mitigate blood–brain barrier damage, suppress inflammatory responses, and decrease cerebral blood flow (26). In a study conducted by Chang et al., the neuroprotective properties of Dex were reaffirmed. Their findings indicated that administering low-dose Dex via pump injection during and after surgery could lower the occurrence of CHS within a three-day postoperative period in individuals undergoing intracranial carotid artery stenting (27). Our study examined AIS patients, monitoring CHS incidence within 7 days post-operation, as this is a critical period for CHS onset (28). However, patients receiving Dex reported lower NRS scores for postoperative pain. Thus, we suggest that Dex may reduce CHS incidence by mitigating pain, aligning with findings from Chang et al. (27). Furthermore, our study indicates that postoperative blood pressure in patients administered Dex was lower more, yet remained within the normal range. This could explain why Dex reduces the occurrence of postoperative CHS in AIS patients. Postoperative hypertension is strongly linked to CHS. In AIS patients, impaired cerebral autoregulation in ischemic or infarcted areas can result in increased cerebral blood flow and CHS due to acute rises in cerebral perfusion pressure from arterial hypertension or revascularization (29–31). Our study observed that postoperative blood pressure in patients receiving Dex was lower than in the placebo group, yet remained within the normal range, with a reduced incidence of postoperative sinus tachycardia. This aligns with findings by Su et al. (13). Dex’s high selectivity for *α*-2 adrenergic receptors allows it to act on sympathetic nerve terminals, decreasing norepinephrine release and enhancing vagal activity (32). These mechanisms enable Dex to regulate the autonomic and cardiovascular systems, providing neuroprotective effects and reducing the incidence of postoperative CHS in AIS patients. A retrospective study utilizing arterial spin labeling MRI revealed a 48% incidence of CHS in patients with AIS following successful revascularization (4). Our study indicates that the low incidence of CHS may result from two factors. First, Dex’s analgesic effect might have obscured symptoms in patients with mild CHS manifestations. Second, by prioritizing non-invasiveness, repeatability, and cost-effectiveness, we monitored the MFV of the MCA using TCD to detect cerebral hyperperfusion, instead of conducting postoperative cerebral perfusion imaging. This approach may have also overlooked some CHS-positive cases.

Unfortunately, our study shows the administration of Dex did not produce a significant impact on the postoperative mRS score, NIHSS score, overall hospitalization duration, ICU stay duration, or the 30-day all-cause mortality rate of the patients. The limited sample size in this study may account for the observed outcomes, as the sample size calculation focused solely on the incidence of CHS. The incidence of postoperative sinus tachycardia in patients treated with Dex was low, with no significant differences in other adverse events, suggesting the regimen’s safety due to minimal hemodynamic effects. This aligns with findings by Su et al. (13). Nonetheless, the study is subject to several constraints. Primarily, the sample size is limited, and further multicenter studies are required to expand the sample size to investigate the impact of Dex on long-term neurological outcomes in patients with AIS after surgery, which also represents the direction of our subsequent research. Furthermore, Dex was not administered intraoperatively in this investigation. Future research could explore preemptive low-dose Dex administration pre-, intra-, and post-anesthesia to assess potential enhanced clinical outcomes. Lastly, all participants underwent general anesthesia, necessitating further examination of individuals undergoing local anesthesia during surgery.

In conclusion, our study suggests that administering low-dose Dex via pump infusion before anesthesia induction and within 72 h post-operation can effectively decrease the occurrence of CHS within 7 days post-operation in AIS patients and alleviate postoperative pain. Nonetheless, broader research is warranted prior to widespread implementation.

## Data Availability

The original contributions presented in the study are included in the article/Supplementary material, further inquiries can be directed to the corresponding author.
