# Supplementary material for: Dexmedetomidine decreases cerebral hyperperfusion syndrome incidence following mechanical thrombectomy in acute ischemic stroke: a double-blind, randomized controlled trial
Source: Front Neurol. 2025 Sep 17;16:1680268. doi: 10.3389/fneur.2025.1680268 (PMC12483894; doi:10.3389/fneur.2025.1680268)
Supplement: Supplementary file 2 [file Table_2.DOC]

Informed Consent Form for Medical Scientific Research Patients

of Civil Aviation General Hospital

Title: Effect of general anesthesia on prognosis of emergency endovascular mechanical thrombectomy in patients with acute ischemic stroke

Project number：202219

Major investigators：Gao Zi-wen, Zhou Ri-hua, Sang Ben-ling, Gao Guo-lin, Li Shu, Li Jia-xin

**Dear Patient:**

You are cordially invited to partake in the research entitled "Impact of General Anesthesia Participation on the Outcome of Emergency Endovascular Mechanical Thrombectomy in Individuals with Acute Ischemic Stroke." This investigation will be carried out at the Civil Aviation General Hospital, with an anticipated participation of 130 volunteers. The study will comprise two cohorts: the experimental group receiving dexmedetomidine injection and the control group receiving a saline placebo. Your assignment to either group will be randomized.

This consent form furnishes essential information to aid in your decision regarding participation in the clinical study. Your involvement is voluntary and has undergone review by the institution's Ethics Committee. Please review this document thoroughly and direct any inquiries to the study's overseeing investigator.

**Drug Profile:**

Dexmedetomidine hydrochloride injection primarily comprises dexmedetomidine hydrochloride, chemically known as (+)-4-(S)-[1-(2,3-dimethylphenyl) ethyl]-1H-imidazole hydrochloride. This medication is indicated for: (1) sedating non-intubated patients pre- and intra-operatively; (2) sedating surgical patients under general anesthesia during endotracheal intubation or mechanical ventilation; and (3) sedating patients on mechanical ventilation in intensive care settings. It acts as a relatively specific agonist of the α2-adrenergic receptor, inducing sedation.

**Research Background：**

Acute ischemic stroke is a condition characterized by significant morbidity, disability, and mortality rates. The primary treatment modalities for this condition involve timely recanalization, which includes intravenous thrombolysis and mechanical thrombectomy. The irreversible damage to the central nervous system resulting from short-term ischemia and hypoxia underscores the critical importance of prompt recanalization within a specific time frame. Additionally, ischemia-reperfusion injury subsequent to vascular recanalization poses further challenges to patient prognosis. Cerebral ischemia-reperfusion injury manifests as a rapid cascade of events, encompassing disruptions in energy production, inflammatory responses, release of excitatory amino acids, dysregulation of calcium ion channels, generation of oxygen free radicals, activation of apoptosis genes, among others. These interconnected processes culminate in a detrimental cycle leading to degeneration, necrosis, or apoptosis of brain cells.Thus, beyond addressing vascular recanalization, therapeutic strategies must also target the cascade of events leading to neuronal death, known as neuroprotective treatment. Dexmedetomidine, by reducing cerebral blood flow and exhibiting neuroprotective properties, has shown promise in animal models of ischemia/reperfusion injury through mechanisms involving the inhibition of these cascades. Consequently, dexmedetomidine represents a potential novel approach for neuroprotection in such patients. Given the limited number of prospective randomized controlled clinical trials investigating the neuroprotective effects of dexmedetomidine in patients with acute ischemic stroke globally, we undertook this study to further elucidate its potential in patients with acute anterior circulation occlusion due to ischemic stroke, aiming to enhance patient outcomes.

**Research Purpose：**

In the present study, eligible patients with acute anterior circulation occlusive stroke will be prospectively enrolled. A comparative analysis will be performed between patients receiving perioperative dexmedetomidine and those in a blank control group to investigate outcomes following endovascular mechanical thrombectomy for acute ischemic stroke.​Evaluations will include the National Institutes of Health Stroke Scale (NIHSS) scores immediately after thrombectomy. Serial blood samples will be collected on postoperative days 1, 3, and 7 to measure levels of interleukin-6, tumor necrosis factor-α, and S100 protein. The duration of hospital stay will also be recorded, and functional outcomes will be assessed using the modified Rankin Scale (mRS) at 30 and 90 days post-discharge.​This research seeks to determine the potential efficacy of dexmedetomidine in improving prognoses of patients undergoing endovascular thrombectomy for acute ischemic stroke, while exploring its underlying mechanisms of action. The anticipated findings aim to provide valuable evidence-based insights to inform clinical practice.

**Process and Methods of the Research：**

This clinical study is divided into the following phases: immediate screening (Day 0), immediate baseline assessment (Day 0), anesthesia induction phase (Day 0), and follow-up period (postoperative Days 1, 3, and 7; 30 and 90 days after discharge). After signing the informed consent form, subjects will immediately undergo screening examinations and assessment against the inclusion/exclusion criteria. Eligible subjects identified during screening will undergo a brief re-evaluation of inclusion/exclusion criteria and completion of baseline measurements during the baseline phase. Upon confirmation of eligibility, subjects will be randomized into study groups.​ In the experimental group, dexmedetomidine injection will be administered as an intravenous infusion at a dose of 0.5 μg/kg over a period exceeding 10 minutes prior to anesthesia induction. Following awakening from anesthesia and before leaving the operating room, subjects in the experimental group will receive a continuous infusion of dexmedetomidine injection at 0.2 μg/kg/h for 24 hours. For the control group, an equal volume of normal saline will be administered via intravenous infusion at the same time points and for the same duration as in the experimental group.​ Throughout the study period, physicians will closely monitor and record various efficacy and safety indicators, including details of study drug administration, serum levels of interleukin-6, tumor necrosis factor-α, and S100β protein on postoperative Days 1, 3, and 7, vital signs, postoperative NIHSS scores, adverse events, and concomitant medications.​

**Risks and Discomforts of the** **Research:**

No serious adverse events related to the study drug Dexmedetomidine Injection have been reported. However, any drug may cause discomfort, and a small number of patients using dexmedetomidine injection may have symptoms such as hypotension and bradycardia. If you experience any suspicious symptoms during the course of medication, please tell your doctor and he or she will give you appropriate treatment or guidance.

**Possible Benefits of the Research:**

Through the use of experimental drugs, it is possible to reduce the incidence of cerebral hyperperfusion syndrome after endovascular mechanical thrombectomy in patients with acute ischemic stroke, reduce cerebral ischemia-reperfusion injury, reduce the number of days in the intensive care unit, and improve patient prognosis.

**Other Treatment Interventions:**

There were no interventions other than participation in the study.

**Privacy Issues:**

If you decide to participate in this study, your participation in the trial and your personal data in the trial will be kept confidential. For you, all information will be confidential and your name will not appear in publications and reports. Information that identifies you will not be disclosed to anyone outside of the research team unless you give your permission.

**Costs and Compensation:**

During the study, you should pay for all tests (except for tumor necrosis factor-a and S100 protein laboratory tests).

During the study period, active and free treatment will be given to the injury caused by adverse events caused by participation in this clinical study, and corresponding economic compensation will be given to the patient for the transportation and loss of work caused thereby.

**Voluntary Participation and Withdrawal:**

You have the option to decline participation in this trial or withdraw from it at any time after informing the researcher without facing discrimination or retaliation. Your medical treatment and rights will not be affected as a result. The research physician may terminate your participation in this study if you require other treatments, fail to adhere to the research plan, sustain an injury related to the trial, or for any other reasons.

You can keep informed of the information and research progress related to this study at any time. If you have any questions regarding this trial, or if you experience any discomfort or injury during the research process, or if you have questions about the rights and interests of participants in this study, you can contact Gao Zi-wen at 15600386835.

**Informed Consent Signature:**

I have read this informed consent form. My doctor (signature) has provided me with a detailed explanation of the purpose, content, risks, and benefits of this clinical trial. The doctor has also answered all my questions. I understand this clinical study and voluntarily participate in this research.

Subject (signature): Date:

Contact number:

Legal representative (signature): Date:

Contact number:

Researcher (signature): Date:

Contact number:

(*Note: If the subject is illiterate, a witness's signature is required; if the subject is incapacitated, the consent of an agent is necessary)*
